# Elevated Transaminases: Does It Always Warrant a Liver Biopsy? Lessons Learned From Pompe Disease

**DOI:** 10.1002/jmd2.70050

**Published:** 2025-11-24

**Authors:** Alicia Khazzeka, Rebecca L. Koch, Jeong‐A. Lim, Jeremy D. Ward, Jessica Doxey, William R. Jeck, Priya S. Kishnani

**Affiliations:** ^1^ Division of Medical Genetics, Department of Pediatrics Duke University School of Medicine Durham North Carolina USA; ^2^ Department of Pathology Duke University Durham North Carolina USA

**Keywords:** creatine kinase, GAA enzyme, gene sequencing, liver biopsy, Pompe disease, transaminases

## Abstract

Pompe disease (PD) is an autosomal recessive disorder caused by pathogenic variants in *GAA*, resulting in acid alpha‐glucosidase (GAA) deficiency and lysosomal glycogen accumulation. PD is classified into infantile‐onset (IOPD), characterized by cardiomyopathy and death within the first year if untreated, and late‐onset (LOPD), which presents with gradual muscle weakness at variable ages. Incidentally elevated transaminase levels are common in LOPD and reflect muscle injury rather than liver damage. Creatine kinase (CK) levels are often elevated too, further indicating a myopathic origin. However, these elevated transaminases may be misattributed to liver disease, prompting a liver biopsy. Three charts of patients who underwent liver biopsies prior to a LOPD diagnosis were reviewed, including clinical presentations and diagnostic workups. Liver histology from these biopsies was evaluated and compared to liver pathology in a GAA knockout mouse model. All cases had elevated transaminases and underwent a liver biopsy. One biopsy was normal, while two showed non‐specific hepatocyte glycogen accumulation. In all cases, aspartate transaminase (AST) levels were higher than alanine transaminase (ALT) levels and CK levels were elevated. Enzyme testing demonstrated GAA deficiency, and genetic testing identified biallelic variants in *GAA*, confirming the diagnosis. These cases highlight the importance of meticulous phenotyping before liver biopsy. A muscle origin should be considered when AST:ALT > 1 with elevated CK levels. Neuromuscular gene panels and GAA enzyme testing offer a non‐invasive diagnostic approach in LOPD. Notably, even when glycogen accumulation is observed in the liver histologically, liver disease is not associated with PD.

## Synopsis

LOPD is a neuromuscular glycogen storage and lysosomal disorder. In the setting of elevated transaminases with AST:ALT ratio > 1 and elevated CK levels without other signs of liver involvement, a muscle rather than liver origin is likely, supporting the need for careful phenotyping and consideration of genetic testing before pursuing an invasive liver biopsy.

## 1 Introduction

Pompe disease (PD) (OMIM 232300), also known as glycogen storage disease (GSD) type II, is an autosomal recessive disorder caused by a deficiency of the lysosomal enzyme acid alpha‐glucosidase (GAA). As a result, glycogen accumulates across the body, especially in skeletal, cardiac, and smooth muscles. This results in impaired autophagosomal‐lysosomal fusion, which accelerates the production of lipofuscin deposits and leads to altered muscle architecture [1].

PD is broadly classified into infantile‐onset (IOPD) or late‐onset Pompe disease (LOPD) based on the level of residual GAA enzyme activity. Patients with IOPD present in the first days to weeks of life with hypotonia and progressive cardiomyopathy, causing death before the age of 2 years if untreated. Patients with LOPD can present from the first year of life to the 6th decade with varying degrees of severity. The primary concern in LOPD is progressive skeletal and respiratory muscle weakness [2].

In addition to genetic sequencing of *GAA* and enzyme testing of GAA in blood‐based assays, skin fibroblasts, or muscle tissue, blood and urine‐based biomarkers can support a diagnosis of PD and aid in patient management. These include elevated urine glucose tetrasaccharide (Glc4) levels and elevated blood levels of creatine kinase (CK), lactate dehydrogenase (LDH), aspartate transaminase (AST), and alanine transaminase (ALT) [3, 4]. Elevated transaminase levels in LOPD are typically the result of muscle damage. In addition to the liver, AST is produced by skeletal and cardiac muscles, the brain, and the kidneys, whereas ALT is predominantly expressed in the liver, with less (albeit still significant) concentrations in muscle tissue [5]. As such, the AST:ALT ratio is used to differentiate between hepatic and non‐hepatic causes. An AST:ALT ratio > 1 usually requires a broader differential diagnosis, including muscle and cardiac disease, especially in the absence of signs of liver injury [5, 6]. We present three molecularly confirmed cases of LOPD that underwent a liver biopsy due to persistently elevated blood transaminase levels. In retrospect, they all had elevated AST: ALT ratios with elevated CK levels, suggesting muscle involvement rather than liver disease. Moreover, the liver histology from a GAA knockout mouse model was evaluated to further contextualize the findings.

## 2 Methods

### 2.1 Patient Data

A retrospective search of the medical records of three cases with LOPD who had a liver biopsy as part of a workup of persistent transaminitis was conducted. Patient data, including liver pathology reports, was obtained, and available liver biopsy slides (Patient 3 only) were reviewed in accordance with Duke Health Institutional Review Board (IRB) policies.

For the pathologic interpretation of the original liver biopsies, liver pathologists (J.D.W. and W.R.J.) independently reviewed the original liver biopsy slides from Patient 3 (Table S1) and compared the diagnoses with the previously rendered pathologic report in the medical record. A microscopic description and interpretation were written for the study after being agreed upon by both pathologists. The pathologists were not blinded to the patient's original diagnosis and utilized any relevant information within the medical record to interpret the liver biopsy.

### 2.2 Mouse Model, Tissue Collection, and Histopathology

All animal procedures were approved by the Duke University Institutional Animal Care and Use Committee (IACUC) and conducted in accordance with National Institutes of Health (NIH) guidelines for the care and use of laboratory animals. C57BL/6J wild‐type (WT) and GAA knockout (KO) mice (B6;129‐Gaatm1Rabn/J) [7] were obtained from the Jackson Laboratory. WT and KO mice were weighed and sacrificed after 24 h of fasting. The whole liver and heart were collected, and their weights were recorded to calculate liver‐to‐body and heart‐to‐body weight ratios, at three time points (3, 6, and 12 months). The heart‐to‐body weight ratio of these mice was used as a positive control. Liver samples from the left lobe were fixed in 10% neutral‐buffered formalin for 48 h at room temperature, followed by post‐fixation in 1% periodic acid in 10% neutral‐buffered formalin for 48 h at 4°C. Then, samples were washed with PBS, dehydrated through a graded ethanol series, cleared with xylene, and embedded in paraffin.

Paraffin‐embedded, formalin‐fixed liver sections were cut using a microtome and stained with periodic acid‐Schiff (PAS) as previously described [8]. Briefly, slides were deparaffinized, rehydrated, and oxidized with freshly prepared 0.5% periodic acid for 5 min, then rinsed with distilled water for 1 min. Slides were stained with Schiff reagent for 15 min, washed with tap water for 10 min, and counterstained with hematoxylin for 3 min. After a final rinse with tap water, slides were incubated with a bluing reagent for 1 min, dehydrated, and mounted. All staining reagents were obtained from Sigma‐Aldrich (St. Louis, MO). Images were acquired using a Keyence BZ‐X710 microscope.

## 3 Results

### 3.1 Case Reports

#### 3.1.1 Patient 1

A 34‐year‐old White male patient first presented to the metabolic genetics clinic at age 30 years. He reported he was always slower than his peers and had a history of thigh muscle weakness despite being athletic. At age 23 years, he required arm support to rise from a chair. At age 24 years, he completed a triathlon and experienced marked shortness of breath and difficulty with running and breathing. In his mid‐20s, following an intercurrent illness, he was found to have persistently elevated transaminases (AST: 114 U/L, ALT: 70 U/L, reference range: AST:8–84 U/L, ALT:15–47 U/L), which prompted a liver biopsy that showed no abnormalities (Table 1). Upon further workup, given his history of difficulty with running, CK levels were assessed and found to be elevated (1002 U/L, reference range: 80–354 U/L). An electromyogram (EMG) was performed and was unremarkable. On subsequent neuromuscular evaluation, he was noted to have persistently elevated CK levels, subtle calf hypertrophy, but no evident weakness. A gene panel for neuromuscular disorders revealed biallelic pathogenic variants in *GAA*: c.‐32‐13T > G and c.525delT. This, in addition to blood GAA enzyme levels in the deficiency range, led to a diagnosis of LOPD at age 29 years.

**TABLE 1:** Characteristics and liver biopsy results from three patients with LOPD.

ID	Sex	*GAA* variants		GAA enzyme activity	AST (U/L)	ALT (U/L)	AST:ALT ratio	CK (U/L)	Liver biopsy findings					
		Allele 1	Allele 2						Age (years)	Glycogen accumulation	Steatosis	Fibrosis	Iron deposition	Overall impression
1	M	c.‐32‐13T > G	c.525delT	Leukocytes: 2.35 (5.98–15.5 nmol/h/mg)	114	70	1.6	1002	29	−	−	−	−	Nondiagnostic
2	F	c.[1477C > T;2221G > A]	c.1978C > T	Blood: 0.07 (0.22–0.62 μmol/min/g)	264	150	1.8	1529	5	+	−	−	−	Consistent with GSD
3	M	c.‐32‐13T > G	c.525delT	DBS: 2.3 (> 3.88 pmol/punch/h)	190	144	1.3	1105	14	+	−	−	−	Consistent with GSD

*Note:* +, present; −, not present. GAA enzyme activity reference range is indicated in parentheses.

Abbreviations: ALT, alanine transaminase; AST, aspartate transaminase; CK, creatine kinase; DBS, dried blood spot; F, female; GAA, acid alpha‐glucosidase; GSD, glycogen storage disease; M, male; U/L, unit per liter.

Shortly thereafter, and as part of LOPD monitoring, a pulmonary function test (PFT) was performed in both supine and upright positions. The results showed normal forced vital capacity (FVC, 105% predicted), total lung capacity (TLC), and maximum voluntary ventilation, with preserved diffusing capacity and no restrictive physiology. He was initiated on enzyme replacement therapy (ERT) with alglucosidase alfa at a dose of 20 mg/kg every 2 weeks at age 29 years. His first physical therapy (PT) assessment at 30 years old showed mild proximal hip weakness (hip flexion 4− 4+/5, extension 4/5) and tightness in the hamstrings (70° passive motion bilaterally, reference range < 30°) but otherwise preserved function (Gait, Stairs, Gower's, Chair Scale (GSGC) 4/27, Quick Motor Function Test (QMFT) 64/64). His six‐minute walk test (6MWT) was 751 m (95% predicted), within the normal range, and he exhibited no gait abnormalities. His urine Glc4 was 2.4 mmol/mol creatinine (reference range: < 4 mmol/mol creatinine) at age 30 years. He remains clinically stable on this ERT regimen, and his most recent labs at age 33 years showed normal urine Glc4 0.7 mmol/mol creatinine (reference range: < 4 mmol/mol creatinine), normal AST 36 U/L, and ALT 45 U/L (reference range: AST:8–84 U/L, ALT:15–47 U/L), and mildly elevated CK 405 (U/L reference range: 80–354 U/L). At his most recent PT assessment, at 31 years of age, he reported he is in better athletic shape than in his 20s. The assessment also demonstrated persistent mild proximal weakness (hip flexion 4− 4+/5, extension 4/5). He had persistent tightness in the hamstrings (70° passive motion bilaterally, reference range < 30°). His 6MWT decreased modestly to 607 m (78% predicted) with otherwise stable function (GSGC 4/27, QMFT 64/64).

#### 3.1.2 Patient 2

A 22‐year‐old Filipina female first came to medical attention at age 5 years with poor weight gain, easy fatigability, elevated transaminases, and elevated CK levels. Her mother reported that she seemed clumsier and fatigued more quickly than her peers, frequently tripping and requiring frequent breaks after short walks. She also had persistent abdominal pain and diarrhea, and was evaluated for celiac disease, which was negative. Due to elevated transaminases (AST: 264 U/L, ALT: 150 U/L, reference range: AST:10–50 U/L, ALT:10–40 U/L), a liver biopsy was performed at age 6 years and revealed nonspecific glycogen accumulation within the hepatocytes and no other abnormalities. Elevated CK levels (1529 U/L, reference range: 80–250 U/L) prompted a muscle biopsy which revealed increased glycogen stores, with distended and distorted fibers containing vacuoles, and decreased GAA activity, suggesting a diagnosis of LOPD. Genetic testing of *GAA* confirmed the diagnosis, identifying the likely pathogenic variant c.1477C > T, and the pathogenic variant c.1978C > T in trans with a variant of uncertain significance c.2221G > A (Table 1). At age 6 years, ERT was initiated with alglucosidase alfa 20 mg/kg every 2 weeks.

At 9 years of age, a baseline PT assessment was conducted which showed diffuse hypotonia and proximal weakness, particularly involving neck flexors (2−/5) and shoulder flexors/abductors (3+–4−/5), with postural abnormalities including anterior pelvic tilt. She had scapular winging, hypermobility, and mild genu valgus. On functional testing, she required hand support to rise from the floor through half‐kneeling and had difficulty jumping and hopping, consistent with weakness of proximal muscles. The 6‐min walk test (6MWT) measured 389.9 m (below the expected mean 483 ± 40 m), and she fatigued after moderate exertion. Her GSGC was 6/27. In addition, PFT was performed which demonstrated upright FVC 57.4% predicted, consistent with moderate restrictive lung disease, and she was concurrently diagnosed with obstructive sleep apnea and asthma. Supine testing results were not available. Urine Glc4 levels were elevated (16.6 mmol/mol creatinine, reference range: < 4.4 mmol/mol creatinine). Based on these findings, her dose of alglucosidase alfa was increased to 40 mg/kg/week. She continued on this ERT regimen through adolescence and demonstrated gradual functional improvement. At age 19 years, she was switched to avalglucosidase alfa at 40 mg/kg every 2 weeks, resulting in marked improvement in muscle strength, resolution of clumsiness, and sustained absence of fatigue. She remains clinically stable on this ERT regimen at 21 years of age with normal urine Glc4 3.3 mmol/mol creatinine (reference range: < 4.4 mmol/mol creatinine), normal AST 20 U/L and ALT 17 U/L (reference range: AST:10–50 U/L, ALT:10–40 U/L), and normal CK 319 U/L (reference range: 80–250 U/L). Her most recent PT evaluation at 21 years of age showed that her 6MWT was 334 m (43% predicted), reflecting reduced endurance compared to peers but preserved stability and posture. Strength testing demonstrated stable proximal muscle performance (GSGC unchanged, QMFT 45/64), and her primary limitations involved flexibility, endurance, and balance rather than overt weakness. At that time, she was using power mobility only for long‐distance community navigation.

#### 3.1.3 Patient 3

**FIGURE 1:**
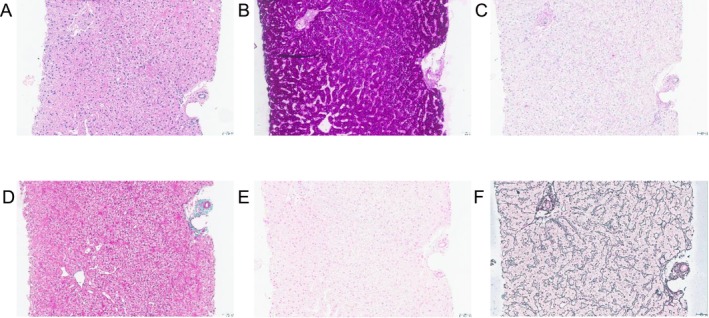
Representative histology of a liver core biopsy from a patient with LOPD (Patient 3). (A) On H&E, the hepatocytes demonstrated an abnormally pale and rarified cytoplasm, with conspicuous cell membranes, and small, hyperchromatic nuclei. (B) PAS staining of the biopsy demonstrated a diffuse accumulation of PAS‐positive granules within the cytoplasm of the hepatocytes. (C) These PAS‐positive granules were absent when the biopsy tissue was pretreated with diastase and then stained with PAS (PAS‐D), consistent with glycogen deposition within the cytoplasm of the hepatocytes. No steatosis was noted within the hepatocytes. (D) The Masson's trichrome stain was negative for fibrosis. (E) The iron stain was negative for intrahepatic or iron deposition. (F) The reticulin stain demonstrated normal parenchymal architecture with no evidence of abnormal nodule formation. All images were taken at 200× magnification.

A 23‐year‐old White male patient had persistently elevated transaminase levels and CK levels beginning at age 7 years. His parents reported that he had normal childhood development and had no trouble keeping up with his peers while playing soccer. He did not complain of muscle weakness or muscle aches. He was referred to neurology for assessment of persistent elevated CK levels at age 7 years. At the time, the neurologist noted the absence of significant weakness or limitations in day‐to‐day activities and deferred further evaluation, while still following up on a yearly basis. At age 15 years, he still had persistently elevated transaminases (AST: 190 U/L, ALT: 144 U/L, reference range: AST: 10–40 U/L ALT: 7–56 U/L) and elevated CK levels (1105 U/L, reference range: 22–198 U/L). Evaluation by a gastroenterologist led to a liver biopsy, which revealed pale lobular parenchyma and fluffy hepatocytes with glycogen accumulation (PAS‐positive granules) (Figure 1). Given the elevated CK levels and the presence of glycogen in the liver, blood GAA enzyme activity was assessed and found to be deficient. This was consistent with an elevated urine Glc4 level at 6.5 mmol/mol creatinine (reference range: < 3.0 mmol/mol creatinine). *GAA* sequencing confirmed the diagnosis of LOPD with pathogenic variants c.‐32‐13T > G and c.525delT (Table 1).

Following the LOPD diagnosis, a baseline PT evaluation at age 15 years revealed mild bilateral hip weakness (4/5) without functional limitation. Gait analysis showed a tendency toward flat‐foot gait, and mild postural asymmetry, with decreased flexibility in the left hamstrings, quadriceps, and gastrocnemius muscles. His 6‐min walk test (6MWT) was 718 m (86% of predicted), and he scored normal (4/27) on the GSGC and low (97.8%) on the Gross Motor Function Measure (GMFM), reflecting subtle proximal weakness and reduced endurance compared to age‐matched peers. PFT at age 16 years was within normal limits and repeat PFT at 23 years demonstrated FVC 97% of predicted with an 18% positional decline in FVC, indicating stable lung mechanics and preserved respiratory strength compared with prior testing. As of the most recent follow‐up at age 23 years, he remains off ERT due to patient preference.

### 3.2 GAA KO Mouse Model Liver Histology

**FIGURE 2:**
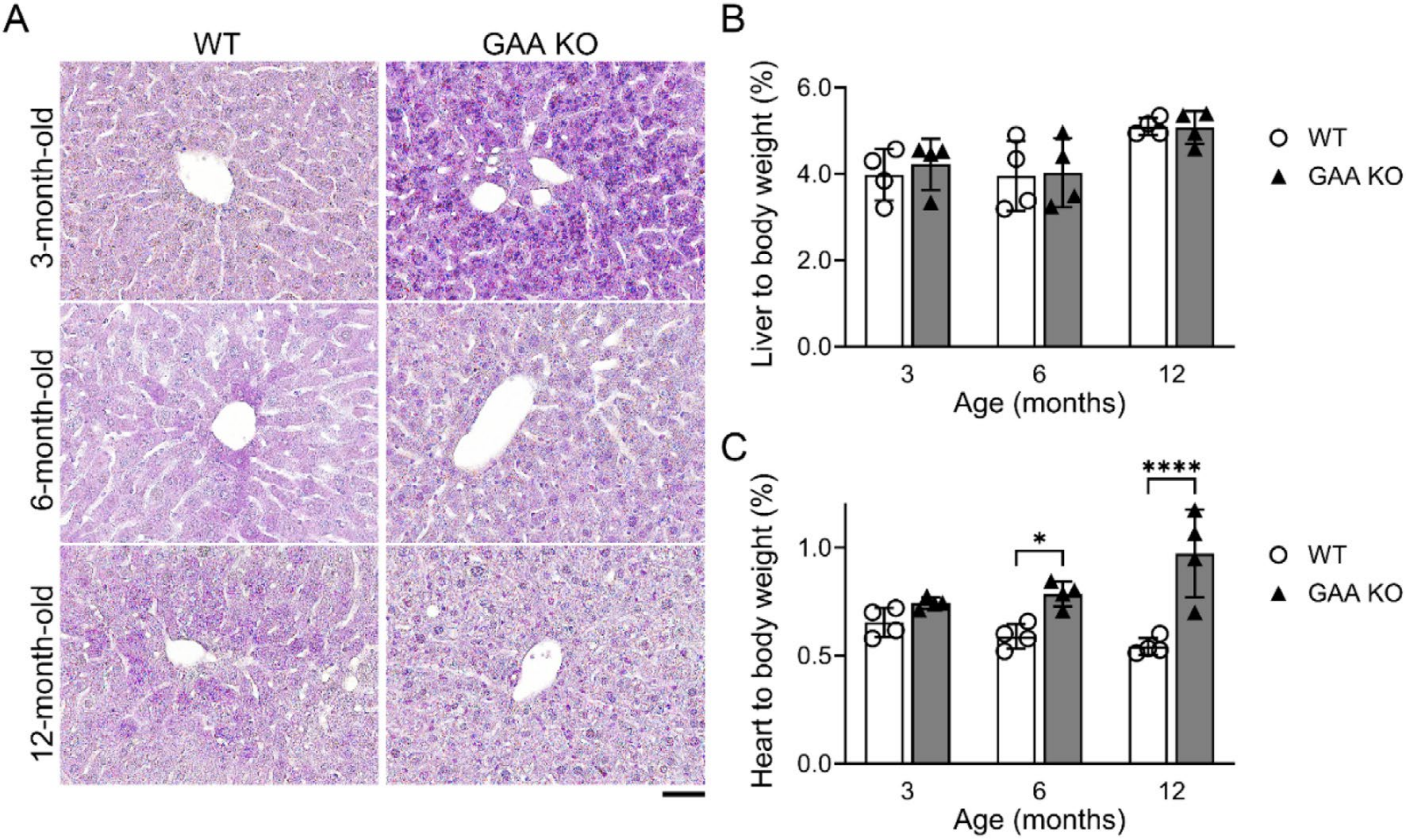
GAA KO mouse liver size and histopathology. (A) Periodic acid‐Schiff staining (PAS, glycogen staining) was performed using paraffin‐embedded liver sections from GAA KO mice at 3, 6, and 12 months of age. Glycogen accumulation, purple dots, in the lysosome was observed in the liver of 3‐month‐old GAA KO mice; however, this accumulation was not detectable in 6‐ and 12‐month‐old mice. Age‐matched WT mice were used as WT controls, and all mice were fasted for 24 h prior to sacrifice. The scale bar represents 100 μm. (B) Liver and (C) cardiac size to assess for hepatomegaly and cardiomegaly were evaluated by calculating the liver‐to‐body weight and heart‐to‐body weight ratios, respectively. The liver size was not significantly different compared to age‐matched WT, while the heart size was increased in GAA KO mice after 6 months of age. Each dot represents one mouse, and the data are presented as mean (SD). Statistical analysis was performed using two‐way ANOVA, with significance denoted by *p < 0.05 and ****p < 0.0001.

Histological analysis using PAS staining revealed distinct glycogen accumulation in the livers of 3‐month‐old KO mice, which was absent in age‐matched WT controls (Figure 2A). This glycogen accumulation was no longer seen in KO mice at 6 and 12 months of age. Despite the early histologic changes, liver‐to‐body weight ratios did not significantly differ between KO and WT mice at any time point (Figure 2B). At 3 months, KO mice showed a slightly lower mean liver‐to‐body weight ratio compared to WT, with similar patterns at 6 months and 12 months, none of which reached statistical significance, indicating an absence of hepatomegaly. In contrast, heart‐to‐body weight ratios demonstrated a progressive increase in KO mice relative to WT (Figure 2C). At 3 months, the difference was modest and not statistically significant; however, by 6 months, KO mice exhibited a significantly higher ratio than WT mice (0.91% vs. 0.73%, *p* < 0.05). This trend continued and became more pronounced at 12 months, with KO mice displaying markedly elevated heart size (1.1% vs. 0.78%, ***p < 0.0001), consistent with progressive cardiomegaly associated with GAA deficiency.

## 4 Discussion

The three cases in this article showcase the phenotypic variability and diagnostic complexity in LOPD. All three had long‐standing laboratory abnormalities with elevated CK and transaminase levels, yet their diagnosis was delayed (1–8 years). In two of the cases (Patients 2 and 3), a liver biopsy revealed glycogen accumulation, and while nonspecific, this steered suspicion toward a GSD. In the other case (Patient 1), a liver biopsy was normal, and only after persistent unexplained elevation in transaminases and CK levels did this patient undergo genetic testing and enzyme analysis. This led to a diagnosis of LOPD, reinforcing that liver pathology can be absent in LOPD.

LOPD can be diagnosed from as early as the first year of life to as late as the 6th decade and can have a spectrum of features, ranging from limb weakness, low back pain, or exercise intolerance to incidental findings of elevated CK, AST, and ALT. In addition, many cases of PD are now detected through newborn screening, following its addition to the Recommended Uniform Screening Panel (RUSP) in 2015 and subsequent inclusion in many state‐specific newborn screening programs in the United States. However, many affected individuals born before its inclusion or in a state that was not testing for PD remain undiagnosed [9], delaying time to diagnosis and treatment initiation. Additionally, there is a well‐documented genotype–phenotype variation in LOPD [10, 11]. For instance, despite sharing identical pathogenic *GAA* variants (c.‐32‐13T > G and c.525delT), patients 1 and 3 presented with distinct clinical and histologic features at different ages. Hence, this overall variability in the age of presentation and symptoms often results in repeated evaluations, referrals to various specialists, and potential misdiagnoses. Collectively, these delays in the initiation of treatment contribute to increased morbidity, including fatigue and worsening respiratory involvement [3, 12].

Due to the nonspecific incidental laboratory findings, particularly elevated transaminases, LOPD can be misattributed to liver disease, leading to a liver biopsy. However, a liver biopsy cannot provide a definitive diagnosis of LOPD. Glycogen accumulation in hepatocytes (as seen in patients 1 and 2) does not confirm a specific diagnosis of GSD, and it can be attributed to other diagnoses, such as glycogenic hepatopathy or exposure to certain medications [13]. Moreover, the lack of pathologic findings on liver biopsy (as in patient 1) does not rule out the presence of a GSD.

In line with these observations, autopsy reports of adult patients with LOPD have shown minimal glycogen accumulation or the absence of any other liver abnormalities [14, 15, 16, 17]. Other abnormalities, such as hepatic steatosis, have only been observed in patients with additional metabolic conditions (i.e., obesity) [18]. Indeed, a successful liver transplantation from a 47‐year‐old donor with LOPD has been reported [17]. In this case report, the explanted liver appeared grossly normal without fatty changes, and a wedge biopsy of the donor liver was normal without increased hepatocyte glycogen, fibrosis, or fatty changes. Similarly, we reported here that in the GAA KO mouse model, liver‐to‐body weight ratios and liver histology were similar between WT and GAA‐deficient mice, further suggesting that liver morphology and function remain unaffected.

In clinical practice, CK measurement is not routinely obtained when evaluating elevated transaminases, as these are often associated with liver injury and their release from hepatocytes. This may misdirect clinicians toward gastroenterology referrals and result in a liver biopsy, rather than evaluation for myopathy. Recognizing that both AST and ALT are also produced by muscle, assessing blood CK levels may provide a relatively non‐invasive and accessible first step to help clarify the etiology. If CK levels are also elevated, it may prompt a more detailed history of muscle involvement and yield an earlier, non‐invasive diagnosis of a myopathy. Indeed, conditions such as rhabdomyolysis and McArdle disease (glycogen storage disease type V) have also presented with abnormal “liver function tests” (i.e., elevated transaminases) that were in fact driven by muscle injury [1, 5, 6, 19].

In all three patients, subtle muscle involvement was evident, ranging from mild proximal weakness and calf hypertrophy in patient 1 to early fatigability in patient 2 and minimal endurance limitation in patient 3. Elevated transaminases prompted liver biopsy before consideration of neuromuscular testing, reflecting a diagnostic focus on excluding primary hepatic disease when biochemical abnormalities were first identified. However, in each instance, the AST:ALT ratio was > 1 and CK levels were elevated, a biochemical pattern more consistent with a muscle rather than hepatic source. As such, our cases highlight that in the setting of elevated transaminases (AST:ALT ratio > 1) and CK without signs of liver disease, less invasive measures can be considered before a liver biopsy. A diagnosis of PD is established through detecting deficient GAA enzyme activity in blood‐based assays [20, 21], skin fibroblasts, or muscle tissue [2, 22], or by identifying biallelic pathogenic variants in *GAA*. Therefore, non‐invasive neuromuscular gene panels, whole exome sequencing (WES), or whole genome sequencing (WGS) using blood or saliva may be a first step to evaluate for several different neuromuscular conditions simultaneously [23, 24, 25]. Blood‐based assays can also be used to assess GAA activity more specifically. Performing these less invasive tests first may allow for faster diagnosis and treatment initiation for individuals with PD.

## 5 Conclusion

The cases described here emphasize the importance of careful phenotyping and clinical suspicion for LOPD in patients with unexplained elevated transaminases, characterized by an AST:ALT ratio greater than 1, and elevated CK levels. Muscle‐related symptoms should be carefully assessed before opting for liver biopsy. Neuromuscular gene panels, WES, or WGS, and testing GAA enzyme activity in blood provide a non‐invasive first step in diagnosing LOPD. Continued efforts are necessary to enhance clinician awareness and expand newborn screening programs, thereby improving patient outcomes.

## Author Contributions

A.K. collected data and wrote and edited the manuscript. R.L.K. and J.D. wrote and reviewed the manuscript. J.D.W., W.R.J., and J.‐A.L. created the figures and wrote and reviewed the manuscript. P.S.K. (the corresponding author) supervised the project and reviewed and edited the manuscript.

## Funding

The authors have nothing to report.

## Ethics Statement

This case series was conducted in accordance with the Duke University Health System (DUHS) Institutional Review Board (IRB), which provided approval for all research involving human participants (Approval No. Pro00010830). Written informed consent was obtained from all participants. The collection and evaluation of all protected patient health information were performed in a Health Insurance Portability and Accountability Act (HIPAA)‐ compliant manner. All procedures followed were in accordance with the ethical standards of the responsible committee on human experimentation (institutional and national) and with the Helsinki Declaration of 1975, as revised in 2000 (5). All animal procedures were approved by the Duke University Institutional Animal Care and Use Committee (IACUC) and conducted in accordance with National Institutes of Health (NIH) guidelines for the care and use of laboratory animals.

## Conflicts of Interest

The authors declare that the research was conducted in the absence of any commercial or financial relationships that could be construed as a potential conflicts of interest. R.L.K. and P.S.K. have received grant or funding support from Alnylam Pharmaceuticals Inc. P.S.K. has received research or grant support from Sanofi Genzyme and Amicus Therapeutics. P.S.K. has received consulting fees and honoraria from Sanofi Genzyme, Amicus Therapeutics, Denali, Regeneron and Asklepios Biopharmaceutical (AskBio). P.S.K. is a member of the Pompe and Gaucher Disease Registry Advisory Board for Sanofi Genzyme, the Pompe Disease Advisory Board for Amicus Therapeutics, and the Advisory Board for Baebies. P.S.K. has held equity in Asklepios Biopharmaceuticals and may receive milestone payments related to that equity in the future.

## Supporting information


**Table S1:** Histopathology review of liver biopsy slides from Patient 3.

## Data Availability

The data that support the findings of this study are available from the corresponding author upon reasonable request.
