# Supplementary material for: Elevated Transaminases: Does It Always Warrant a Liver Biopsy? Lessons Learned From Pompe Disease
Source: JIMD Rep. 2025 Nov 24;66(6):e70050. doi: 10.1002/jmd2.70050 (PMC12643788; doi:10.1002/jmd2.70050)
Supplement: Supplementary file 1 — Table S1: Histopathology review of liver biopsy slides from Patient 3. [file JMD2-66-e70050-s001.docx]

| **Stain** | **Type** | **Representative findings** | **General notes** |
| --- | --- | --- | --- |
| Hematoxylin & Eosin (H&E) | Routine stain | Pale, rarified hepatocyte cytoplasm; small hyperchromatic nuclei; no steatosis | Used twice: for baseline and comparison |
| PAS (without Diastase) | Histochemical | Diffuse accumulation of PAS-positive granules in hepatocytes | Used alongside PAS-D to differentiate glycogen from other PAS-positive substances |
| PAS-D (PAS with Diastase) | Histochemical | PAS-positive granules absent after diastase digestion, confirming glycogen | Glycogen accumulation |
| Trichrome | Histochemical | Negative for fibrosis | Performed manually to improve fibrosis detection |
| Iron | Histochemical | Negative for iron deposition | No evidence of iron overload |
| Reticulin | Histochemical | Normal parenchymal architecture; no nodule formation | No evidence of early cirrhosis or regeneration nodules |

**Supplementary Table 1: Histopathology review of liver biopsy slides from Patient 3.**

Abbreviations: PAS, Periodic Acid–Schiff; PAS-D, Periodic Acid–Schiff with diastase.
